# Bacterial meningitis in a patient with multiple sclerosis receiving Tysabri

**Published:** 2016-07-06

**Authors:** Abdorreza Naser Moghadasi, Soroor Advani, Shiva Rahimi

**Affiliations:** 1 Multiple Sclerosis Research Center, Neuroscience Institute, Department of Neurology, Sina Hospital, Tehran University of Medical Sciences, Tehran, Iran; 2 Department of Neurology, Sina Hospital, Tehran University of Medical Sciences, Tehran, Iran

**Keywords:** Bacterial Meningitis, Multiple Sclerosis, Tysabri

The most important adverse effect of natalizumab is progressive multifocal leukoencephalopathy (PML).^1^ Apart from PML, there are reports of other cerebral infections including herpes simplex encephalitis (HSE)^2^^,^^3^ and cryptococcal meningitis^4^ in the literature. 

The patient was a 29-year-old woman, a known case of multiple sclerosis (MS) for at least 5 years. She was treated using natalizumab since 6 month before. She was under treatment with prednisolone 1 g daily for 5 days for optic neuritis, which was 2 weeks before the onset of symptoms of meningitis. Approximately three days before visiting the neurologist, a continuous headache in the left temporal lobe was developed. The patient was febrile at that time as well. Besides, two days before this, she had started ciprofloxacin for treatment of a urinary tract infection. 

An investigation for the John Cunningham (JC) virus was negative. The brain magnetic resonance imaging (MRI) showed periventricular lesions (Figure 1-A) with no evidence of PML or HSE encephalitis. Meningeal enhancement was seen after the injection of the contrast medium (Figure 1-B).

**Figure 1:**
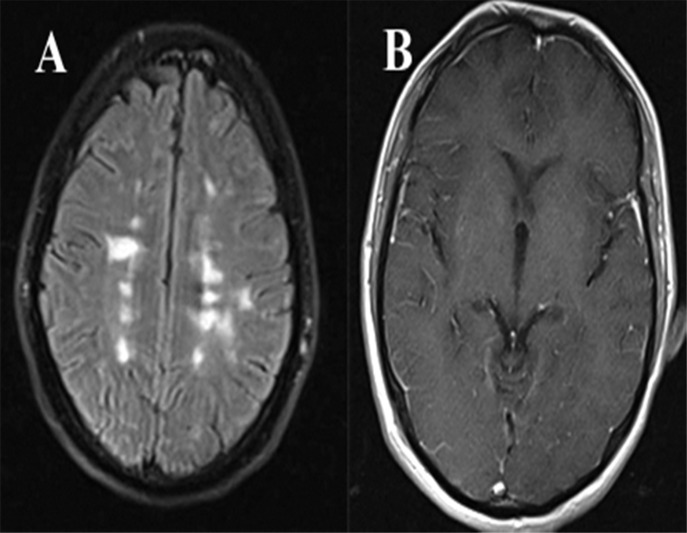
Periventricular lesions confirming the diagnosis of multiple sclerosis (MS) in the FLAIR MRI view (A). Meningeal enhancement was seen after gadolinium injection (B)

The patient underwent a lumbar puncture. Simple analysis of the cerebrospinal fluid (CSF) showed the following: white blood cell (WBC) = 350 (55% lymphocytes and 45% polymorphonuclear cells); red blood cell (RBC) = 20; protein = 150 mg/dl; glucose = 37 mg/dl; and concomitant blood sugar = 155 mg/dl. Other routine tests including blood biochemistry and complete blood cells (CBC) were all normal. 

Considering the patient’s condition and her CSF profile, treatment with ceftriaxone, vancomycin, and acyclovir was started. Since polymerase chain reaction (PCR) assay for HSE-1 and 2 was negative, acyclovir was stopped and other antibiotics were continued with a diagnosis of partially treated bacterial meningitis. The patient’s headache and fever also subsided. 

Other investigations including PCR of the CSF for tuberculosis, cryptococcus, and human immunodeficiency virus (HIV) were all negative.

CSF culture and smear were negative, which could be due to the previous administration of ciprofloxacin. The most probable diagnosis was partially treated bacterial meningitis in consideration of CSF analysis and other investigations as well as the patient’s response to treatment. 

Through targeting the α-4 integrin, natalizumab prevents activated T lymphocytes from entering the brain.^5^ However, despite its marked clinical benefits in patients with MS, there is an occasional cause of fetal adverse effects like PML and HSE.^5^ These mechanisms might have facilitated the development of meningitis in our patients as well.

This case report introduces a different probable complication of natalizumab in a patient with MS. As mentioned above, natalizumab can cause infection in brain and it is not limited to HSE and PML. It should be considered that in every patient receiving any drugs with the potential to alter the immunity of the brain, high suspicion of opportunistic infections is one of the most important points in the patient’s follow-up.^6^

## Notes:

**How to cite this article:** Naser Moghadasi A, Advani S, Rahimi S. Bacterial meningitis in a patient with multiple sclerosis receiving Tysabri. Iran J Neurol 2016; 15(3): 175-6.
